# Colorectal Cancer Screening Challenges in the Recent Afghan Refugee Population: A Comprehensive Review Article

**DOI:** 10.7759/cureus.22400

**Published:** 2022-02-20

**Authors:** Abdul Waheed, Audrey McCloskey, Frank Kennedy, Siamak M Seraj, Jaffar Khan, Noor Nama, Omari Johnson, Peter Lo, Harres Magee, Wazir Akbar, Asad Ullah, Frederick D Cason

**Affiliations:** 1 Surgery, San Joaquin General Hospital, French Camp, USA; 2 Surgery, St. George's University School of Medicine, St. Georges, GRD; 3 Internal Medicine, San Joaquin General Hospital / California Northstate University, French Camp, USA; 4 Pathology and Laboratory Medicine, Indiana University School of Medicine, Indianapolis, USA; 5 Obstetrics and Gynaecology, Bolan Medical College Complex Hospital, Quetta, PAK; 6 General Surgery, San Joaquin General Hospital, French Camp, USA; 7 Medical Education and Simulation, San Joaquin General Hospital, French Camp, USA; 8 Neurology, Bolan Medical College, Quetta, PAK; 9 Pathology, Medical College of Georgia - Augusta University, Augusta, USA

**Keywords:** limitations, colorectal cancer, challenges, screening, afghan population

## Abstract

Colorectal cancer (CRC) is more prevalent in south-central Asian countries, particularly the Afghan population. Screening for CRC in the Afghan population has always been challenging, primarily due to the tribal and social cultures, lack of facilities, and lack of education. The United States (US) will soon face a significantly massive influx of Afghan refugees. It becomes imperative to initiate and implement effective measures regarding CRC screening in these refugee populations. The current review article aims to identify the most likely challenges faced for CRC screening in this Afghan refugee population in the US and address the possible measures to overcome these challenges.

## Introduction and background

The Afghan population is exceptionally prone to gastrointestinal malignancies, with an overall cancer-related age-adjusted mortality rate of 5.13/100,000 in the general population [1]. Screening for CRC is always challenging, and on top of that, screening the refugee population can be very demanding [2]. It is well-known that refugee populations of various ethnic backgrounds deal with even more obstacles to CRC screening than their American-born counterparts [3]. Also, very little is known about CRC screening in the Afghan refugee population, as they mostly get assistance from resettlement organizations momentarily [4].

Moreover, various factors, such as living in a low-income neighborhood, language barriers, immigration status, and not being enlisted in primary healthcare, are associated with reduced CRC screening in refugee populations [4-5]. Although breast cancer is the most frequently reported cancer in the Afghan people, followed by gastrointestinal tract malignancies, screening for CRC is not well-practiced due to cultural and social values [1]. Additionally, recently published data related to the insurance status of the refugees revealed that up to 50% of refugees remain uninsured for the first few years of resettlement and often had reduced access beyond the first eight months [6].

Surprisingly, Afghan refugees have higher health insurance coverage rates than the overall immigrant population [7]. In 2019, just 8% of immigrants from Afghanistan were uninsured, compared to 20% of the total refugee population [8]. Also, Afghan immigrants are more likely to be covered by public health insurance than the overall foreign and US-born populations [7]. Facing the recent massive influx of Afghan refugees in the US, the current review article focuses on determining and addressing the challenges for CRC testing in the refugee population.

## Review

Materials and methods

Literature Search

**Figure 1:**
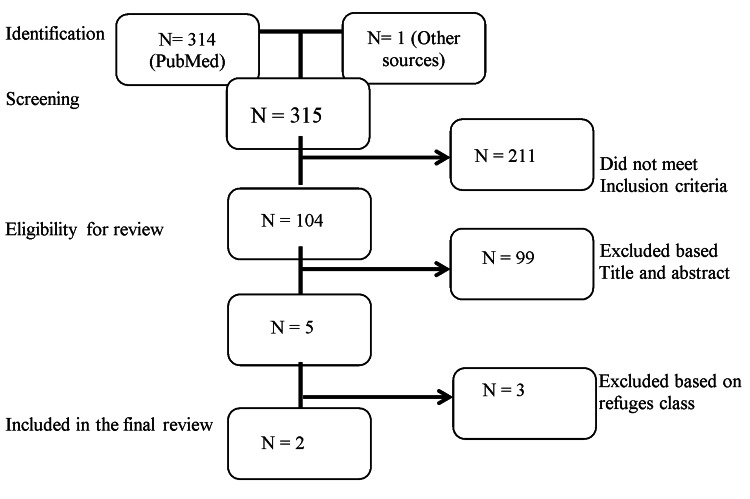
Consolidated Standards of Reporting Trials (CONSORT) diagram Abbreviation: N = Number

An extensive literature search was conducted using PubMed and various national and international conference websites from 1966 to 2021. Only articles published in English were selected for review. Different keywords were used, including refugees, Afghan, immigrants, cancer, colon, rectal, colorectal, screening, and challenges. We mainly focused on findings studies related to colorectal cancer screening in Afghan refugees and excluded studies that did not include colorectal cancer screening (Figure 1).

Articles abstracted were assessed for quality from the internal sources. Three authors of this manuscript evaluated the validity of the included studies. In case of disagreement regarding the study selection, a third author's judgment and agreement were considered.

Quality of Articles

Demographic characteristics of the studies: A total of two studies related to the challenges faced for colorectal cancer screening in the Afghan refugee population were included in the current review. Siddiq et al. interviewed a total of 19 female patients over the age of 50 while Otoukesh et al. included 23,152 participants in their study, comprising 10,997 male participants and 12,155 female participants.

Potential challenges faced in colorectal cancer screening: Based on the data from the studies included in the current review, most of the barriers faced were related to the cultural, educational, social, and accessibility to the healthcare facilities. 

Discussion

Afghanistan has historically been a landlocked country with an estimated population of 32.5 million [1]. Recently, the US has faced a massive influx of Afghan refugees primarily due to regional political instability, and the vast majority of these refugees are settled in California [9-10]. Sacramento County, Fairfax County in Virginia, Alameda County, and Contra Costa County in California accommodate most Afghan refugees in the US [10]. After implementing the US immigration law in 1965, the US has been the international leader in resettling refugees from throughout the globe [11-12]. According to recent data from the United Nations (UN) Department of Financial and Social Affairs, so far >47 million refugees have been settled in the US, which stands for >19% of approximately 244 million global immigrants, and likewise >14% of the US population [13]. With this massive influx of Afghan refugees, assessing CRC screening will pose significant challenges to the US economy and healthcare system.

Furthermore, many obstacles, such as lack of disease expertise, socioeconomic status, time constraints, language obstacles, cultural misconceptions, and perceptions concerning CRC screening, pose added challenges in screening these communities [14]. Likewise, another major challenge the refugee population faces is accessing long-term healthcare assistance to receive routine CRC screening and follow-up care [15]. In a recent study related to the access to the CRC screening by the refugee population, Punzo et al. reported that CRC screening in the refugee population is far less than the native population, which might be due to the lack of access to healthcare facilities [16].

Likewise, CRC screening also depends on the culture and geographical location around the globe [17]. Wang et al., in a mixed-method study of breast and CRC screening barriers, reported that screening programs in countries other than western countries are mainly irregular and heterogeneous [4]. Based on previously published data and the fact that CRC screening behavior varies among people of different origins, developing unique culturally and regional specific screening protocols can be extremely helpful [4].

Additionally, the time spent in the US has been recognized as a substantial forecaster of CRC screening in refugees of different ethnic backgrounds [18-20]. Wong et al. reported that the years settled in the US and English language efficiency was directly correlated to each other, and those refugees who have been living in the US for <15 years are not efficient in the English language [21]. In addition, they reported that those refugees residing in the US for <15 years are about half as probable to have ever gone through CRC screening [21]. Since most Afghan refugees are not fluent in English, as their native language is either Pashto and Persian, we can predict that unfamiliarity with the English language presents an incredible difficulty in understanding the significance of CRC testing in this subset of refugees [22].

The effectiveness of proper language translators and healthcare patient navigators for cancer screening in rural non-Afghan refugee populations has been well-published [23]. Similarly, Afghan refugees will likely benefit from using healthcare patient navigators and proficient translators, easing cultural misconceptions and language barriers, and discussing CRC screening [24-25]. The new influx of Afghan refugees poses an opportunity to address these barriers and better understand the obstacles preventing these populations from participating in long-term screening opportunities, ultimately reducing colorectal cancer-related mortality and overall healthcare costs [26]. This particular group would benefit from developing a culturally unique approach to providing CRC screening services by addressing language barriers and cultural misconceptions [27-28].

Table 1 lists the demography of participants and quality of the identified studies while Table 2 summarizes all the potential challenges faced in the studies included in this review while interacting with the Afghan refugee populations.

**Table 1:** Demography of participants and quality of the identified studies Abbreviation: N = Number; F = Female; M = Male

Study	Year	Participants (N)	Gender	Study design
Siddiq et al. [26]	2020	19	F = 19	Semi-structured interview
Otoukesh et al. [27]	2015	23,152	M = 10,997, F = 12,155	Retrospective, cross-sectional study

**Table 2:** List of barriers to colorectal cancer screening and underlying rationale in the refugee population, particularly in the Afghan community Abbreviation: F = Female

Barriers to Screening Challenges		
Challenges Faced	Rationale	
Older age [26-27]	An increased reluctance of discussion around benefits of colorectal screening	
Gender, F [26-27]	Associated cultural influence in interacting with healthcare workers	
Language barriers [26-27]	English not being the primary language	
Education [26-27]	Limited education	
Type of insurance [26-27]​​​​​​	Lack of insurance on arrival	
Knowledge related to colorectal screening [26-27]	Lack of medical knowledge surrounding benefits of colorectal screening and associated procedures	
Cultural issues [26-27]	Hesitancy related to physical interaction with physicians and healthcare workers mainly associated with the colorectal region	
Societal support [26-27]​​​​​​​	Lack of understanding of sociocultural understanding of refugee communities by local populations.	
Absence of focus groups [26-27]​​​​​​​	Absence of focus groups based on the primary language due to multiple languages within the refugee community.	

Limitation

Although an extensive literature search was performed, our study still has a few limitations. As with most of the database, there might be some studies that we missed during our extensive review on this topic, which may have limited the identification of some challenges that need to be identified. Although all the studies were validated through the interval review process, the external validation of the studies was not performed in this review.

## Conclusions

The Afghan refugee population in the US may be at significant risk for increased rates of colorectal carcinoma due to low levels of CRC screening. Barriers to screening and follow-up among refugee populations are multifactorial and must be better understood to implement new approaches and outreach services. These barriers can be overcome using outreach programs, healthcare patient navigators, translators, and understanding of the various socio-cultural obstacles. The implementation of less invasive testing methods, such as fecal occult blood test (FOBT), may increase adherence to screening in refugee populations, particularly those with access to health insurance. The most recent influx of Afghan refugees presents an opportunity to address these obstacles and provide improved access to essential CRC screening services, reducing related morbidity, mortality, and overall healthcare costs for the US healthcare system.
